# The influence of nanosunflower ash and nanowalnut shell ash on sustainable lightweight self-compacting concrete characteristics

**DOI:** 10.1038/s41598-024-60096-5

**Published:** 2024-04-24

**Authors:** Nahla Hilal, Nadhim Hamah Sor, Marijana Hadzima-Nyarko, Dorin Radu, Taher A. Tawfik

**Affiliations:** 1 Scientific Affairs Department, University of Fallujah, Fallujah, Iraq; 2 Civil Engineering Department, University of Garmian, Kalar, Kurdistan Region Iraq; 3 Department of Civil Engineering, Harran University, Şanlıurfa, Turkey; 4 Josip Juraj Strossmayer University of Osijek, Faculty of Civil Engineering and Architecture Osijek, Osijek, Croatia; 5 Faculty of Civil Engineering, Transilvania University of Brașov, Brașov, Romania; 6 Department of Construction and Building Engineering, High Institute of Engineering, October 6 City, Egypt; 7 Institute of Construction and Architecture, Slovak Academy of Sciences, Dúbravskácesta 9, 845 03 Bratislava, Slovak Republic

**Keywords:** Lightweight self-compacting concrete, Waste plastic, Nano sunflower ash, Nano walnut ash, Elevated temperature, Sulphate attack, Engineering, Civil engineering, Nanoscale materials

## Abstract

The absence of biodegradability exhibited by plastics is a matter of significant concern among environmentalists and scientists on a global scale. Therefore, it is essential to figure out potential pathways for the use of recycled plastics. The prospective applications of its utilisation in concrete are noteworthy. The use of recycled plastic into concrete, either as a partial or complete substitution for natural aggregates, addresses the issue of its proper disposal besides contributing to the preservation of natural aggregate resources. Furthermore, the use of agricultural wastes has been regarded as a very promising waste-based substance in the industry of concrete manufacturing, with the aim of fostering the creation of an environmentally sustainable construction material. This paper illustrates the impact of nano sunflower ash (NSFA) and nano walnut shells ash (NWSA) on durability (compressive strength and density after exposure to 800 °C and sulphate attack), mechanical properties (flexural, splitting tensile and compressive strength) and fresh characteristics (slump flow diameter, T50, V-funnel flow time, L-box height ratio, segregation resistance and density) of lightweight self-compacting concrete (LWSCC). The waste walnut shells and local Iraqi sunflower were calcinated at 700 ± 50 °C for 2 h and milled for 3 h using ball milling for producing NSFA and NWSA. The ball milling succeeded in reducing the particle size lower than 75 nm for NSFA and NWSA. The preparation of seven LWSCC concrete mixes was carried out to obtain a control mix, three mixtures were created using 10%, 20% and 30% NWSA, and the other three mixtures included 10%, 20% and 30% NSFA. The normal weight coarse aggregates were substituted by the plastic waste lightweight coarse aggregate with a ratio of 75%. The fresh LWSCC passing capacity, segregation resistance, and filling capability were evaluated. The hardened characteristics of LWSCC were evaluated by determining the flexural and splitting tensile strength at 7, 14 and 28 days and the compressive strength was measured at 7, 14, 28 and 60 days. Dry density and compressive strength were measured after exposing mixes to a temperature of 800 °C for 3 h and immersed in 10% magnesium sulphate attack. The results demonstrated that the LWSCC mechanical characteristics were reduced when the percentages of NWSA and NSFA increased, except for 10% NWSA substitution ratio which had an increase in splitting tensile strength test and similar flexural strength test to the control mixture. A minor change in mechanical characteristics was observed within the results of LWSCC dry density and compressive strength incorporating various NSFA and NWSA` contents after exposing to temperature 800 °C and immersed in 10% magnesium sulphate attack. Furthermore, according to the findings, it is possible to use a combination of materials consisting of 10–20% NSFA and 10–20% NWSA to produce LWSCC.

## Introduction

Many construction engineers are interested in using self-compacting concrete (SCC) because of its enhanced hardened and fresh characteristics. Lightweight self-compacting concrete (LWSCC) is considered as a combination between demanded lightweight construction material within hardened state and desirable SCC characteristics within fresh form. The use of lightweight aggregate (LWA) as a substitution for normal weight gravel within SCC mixes is considered as a well-known procedure for obtaining LWSCC mixtures^1–4^. However, the lightweight aggregate has a porous characteristic which consumes a large amount of water and provides a stronger matrix of cement that causes a decrement in water/cement ratio. In addition, the lightweight aggregate has the ability to provide non-continuous voids inside the hardened concrete and, therefore, leads to a reduction of the density. However, the usage of Polyvinyl chloride (PVC) as a plastic waste material in the recycling process is recognized as a good solution because of the economic cost and impervious characteristics of PVC^5–9^. An efficient way for disposing of PVC is through the grinding of plastic waste into small particles in order to replace coarse or fine aggregates within concrete mixing. Nevertheless, the use of LWA within LWSCC can lead to a serious issue within the fresh state. Since the matrix phase and LWAs density within LWSCC are obviously different, the LWAs tend to move to the surface of fresh concrete due to the buoyancy force^1^. In order to stop this segregation, specified approaches, such as the increment of the system viscosity, were suggested by different researchers^10,11^ through the use of additional cementitious materials in order to increase viscosity of the matrix. As a result, due to the increase of the content of Portland cement within concrete mix, an increment in the consumptions of energy and environmental pollutions is observed. This impacted the mix durability characteristics within long-term (permeability) and short-term (early age shrinkage cracking). Thus, a part of Portland cement had to be substituted by other powders, such as pozzolanic or inert materials. Also, other materials have been widely approved in previous studies: metakaolin^12^, fly ash^13–16^, waste ceramic powder^17–19^, ground granulated blast furnace slag^20,21^ and eggshell powder^22^, in addition to nano materials as Nano clay^23–26^. Bheel et al.^27^ investigated the impact of using metakaolin as a substitution for fly ash on the transport, mechanical, and fresh characteristics of LWSCC mixes. They found an improvement of mechanical strength by increasing the metakaolin replacement up to 10% and the workability was decreased with increasing of metakaolin content^27^. The impact of waste plastic as fine aggregate on the LWSCC hardened characteristics was studied by Hilal et al.^28^. On the other hand, Ahmed et al.^29^ presented that thermal conductivity reduced with increasing replacement level of recycled waste polypropylene as fine aggregate.

Furthermore, different type of pozzolanic materials can be substituted by ordinary Portland cement to mitigate the technical and environmental issues within LWSCC. The impact of pozzolanic materials, such as waste ceramic powder (WCP) was carried out in a study in conventional vibrated concrete and mortar by several researchers^30,31^. Heidariand and Tavakoli^32^ conducted a study on the impact of replacing concrete specimens with 10–40% of ground ceramic powder. This research illustrated that the ground ceramic addition of up to 20% does not have a significant negative impact on the concrete compressive strength. Moreover, the usage of any ground ceramic quantity within the concrete causes a reduction in the capacity of its water absorption. In addition, many researchers studied the effect of artificial lightweight aggregate on the durability and hardened properties of mortar and self-consolidating concrete^33–35^ and illustrated that the mechanical characteristics decreased. Vejmelková et al.^36^ carried out an investigation on the thermal, durability and mechanical characteristics within the concrete containing ceramic as a replacement for cement and eventually recognized positive outcomes. The remains of ceramic tiles which resulted from polishing procedure were utilized as a supplementary cementing material in conventional cement mortar^30^. The size of measured particle for the ceramic remains was 13.7 μm, which were chemically mainly composed of silicate and aluminate oxides. The mechanical test results illustrated an increment within the compressive strength from the early age of 2 days up to 120 days in all substitution levels (10%, 20%, 25%, 30% and 40%). Also, Hilal et al.^37^ conducted a study on the impact of partial cement substitution to WCP with various weight percentages (0%, 20%, 40% and 60%) on the concrete compressive strength, dry density and workability. Besides, the elevated temperature effect (200–800 °C) at 2 h was also conducted to examine the impact on the concrete residual compressive strength. The results illustrated that the temperature and WCP significantly affected the compressive strength, dry density and workability of the concrete mixtures. However, Aly et al.^38^ investigated the feasibility of producing self-compacting concrete (SCC) mixes yielding acceptable hardened and fresh concrete properties with high-volume WCP inclusion as partial substitution for cement. A slight decrement within the slump flow was observed but with an improvement in other fresh characteristics. Also, the WCP usage slightly causes a decrement in compressive strength. SCC with acceptable characteristics combined with a high-volume WCP were produced. Barnat-Hunek et al.^39^ showed that the hybrid fibers-reinforced LWSCC with perlite aggregate exhibited more ductile behavior than the LWSCC without fibers. During the test of flexural tensile strength, cracks appeared on fibers bridge. Basalt fibres shielded porous LWSCC from the attack of frost successfully, while steel fibers suffered from damage.

However, nanotechnology is from the modern technologies that have significant and worthwhile applications in different industries where the industry of construction is included. The use of nanoparticles causes novel characteristics within concrete which lead to an enhancement in the concrete durability and mechanical characteristics. Because of their pozzolanic reaction and enhancement of impact strength and concrete abrasion, silica nanoparticles reduce the water absorption of the concrete, increase the concrete strength, and help in the formation of denser microstructure within concrete. Moreover, nano TiO_2_ causes self-cleaning characteristics within the concrete. Carbon nanotubes can be utilized as a suitable reinforcement. Also, other nanoparticles such as nano barium silicate, nano barium carbonate, nano Fe2O_3_, nanoAl_2_O_3_, nanofibres, nano clay, and nano silica can be utilized within the construction industry^40–42^. Nano silica is classified as the most usable nanomaterial in improving the performance of concrete^43,44^. In addition, a higher ultrasonic pulse velocity is observed when an additional nano silica quantity is added to the concrete. Adding a sufficient nano silica amount in the concrete matrix, can lead to the following achievements^45,46^: promotion of the cement hydration, enhancement of concrete density, producing C–S–H gel favorable for decreasing of the ITZ (Interfacial Transition Zone) porosity, fill of the pores between cement slurry and C–S–H gel, enhancement of the internal structure, porosity decrease and the control of crystallization degree through the transformation of CH crystals to C–S–H. The usage of nano silica proper dosage caused a formation of dense structure by the NS particles by converting crystals of C–H to C–S–H, thus the cement matrix is determined to be more uniform and compact.

The aim of this research is to examine the influence of nano sunflower ash (NSFA) and nano wheat shell ash (NWSA) as a partial substitution for cement with 0%, 10%, 20%, and 30% levels on lightweight self-compacting concrete (LWSCC) properties with a fixed amount of recycled waste ceramic powder as cement replacement and a fixed amount of waste plastic as a partial replacement of normal coarse aggregate for all mixes. The waste plastic (lightweight coarse aggregate) was made from waste plastic water hose that were gathered and cut manually by special scissors. All LWSCC mixtures were tested for workability regarding the passing capability (L-Box), segregation resistance and filling capability (slump flow diameter, slump flow time (T50) and V-funnel flow time). Moreover, the mechanical characteristics, dry density and compressive strength under elevated temperature and sulfate attack were determined for all mixes.

## Research significance

Iraq is a well-known country for walnut trees and growing sunflower, specifically in the northern regions where the inhabitants use large amounts in manufacturing sweets and pastries which lead to obtaining large amount of crusts. Thus, the recycling of these crusts through the usage in concrete as cement materials aids significantly in producing an environmentally friendly concrete and decreases cement usage. Nevertheless, previous attempts have been made to reuse both walnut shells as coarse or fine aggregate^47–50^ and sunflower ash in micro size^51,52^ within concrete. However, no previous research used walnut shells ash and sunflower ash at nano size. A friendly-environment methodology is needed for facilitating the CO_2_ emissions disposal which resulted from burning of walnut shells and sunflower wastes. In this study, the walnut shells and sunflower are burnt in furnace at 700 ± 50 °C for two hours, in order to convert this waste to ashes and then grinded by a ball mill for three hours. The ground ash is then used as a part of cement modification. Thus, the aim of this research is to utilize the nano sunflower ash (NSFA) and nano walnut shells ash (NWSA) within LWSCC to be used in preventing environmental pollution and saving natural resources within cement industries.

## Materials and methods

### Materials

#### Cement

In this research, the ordinary Portland cement which was utilized is commercially also known as AL-mass. Tables 1 and 2 illustrate the physical characteristics and chemical composition respectively, which conform to ASTM C114^53^.

**Table 1:** The chemical composition of cement, ceramic powder, nano sun flower ash and nano walnut shell ash.

Chemical analysis (%)	OPC	Ceramic	NSFA	NWSA
SiO_2_	68.67	57.40	52.00	45.00
CaO	1.926	5.72	13.00	50.00
MgO	2.49	3.16	3.20	3.38
Al_2_O_3_	0.432	17.98	11.10	6.00
Fe_2_O_3_	1.725	6.20	16.20	1.99
SO_3_	0.758	1.59	0.36	6.55
K_2_O	2.488	4.09	6.37	–

**Table 2:** Fine materials' physical characteristics.

Property	Cement	Ceramic	Nano walnut shell ash	Nano sunflower ash
Specific surface area (cm^2^/gm)	3000	4250	16.535	18.763
Specific gravity	3.15	2.04	2.17	3.20

#### Waste ceramic powder

**Figure 1:**
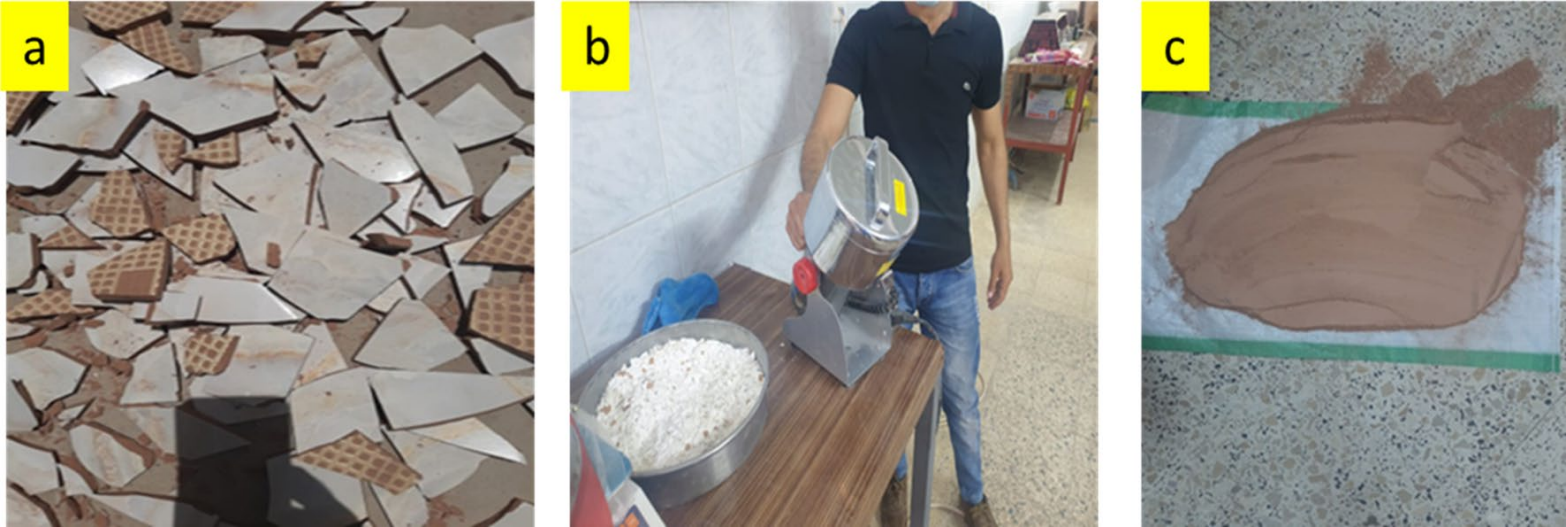
Procedure for preparing the ceramic powder. (**a**) Collecting (**b**) Crushing (**c**) After sieving.

**Figure 2:**
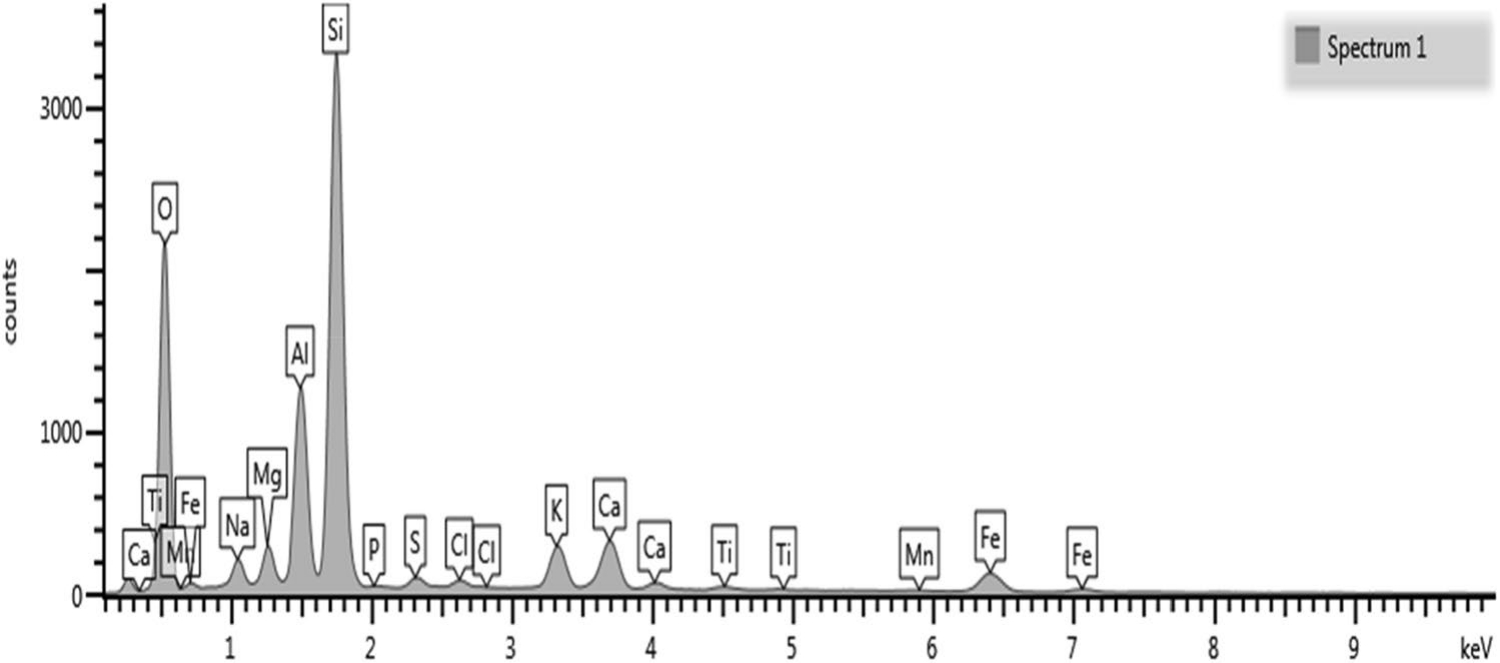
The Spectrum of used ceramic waste in recent study.

In this research, the recovered crushed ceramic waste from Iraqi construction stores was the source of the used waste crushed ceramic. At the laboratory scale, the ceramic powder was acquired by grinding ceramic wastes using an electric mill and then sieved through a 75-µm. Table 1 shows the chemical composition of crushed ceramic, which is depicted in Fig. 1. Furthermore, energy dispersive spectroscopy (EDS) was performed to investigate the existence and microscopic composition of components within waste ceramic powder. Figure 2 depicts the main components of waste ceramic powder, which are aluminum, silicon, and oxygen.

#### Nano sunflower and walnut ashes

**Figure 3:**
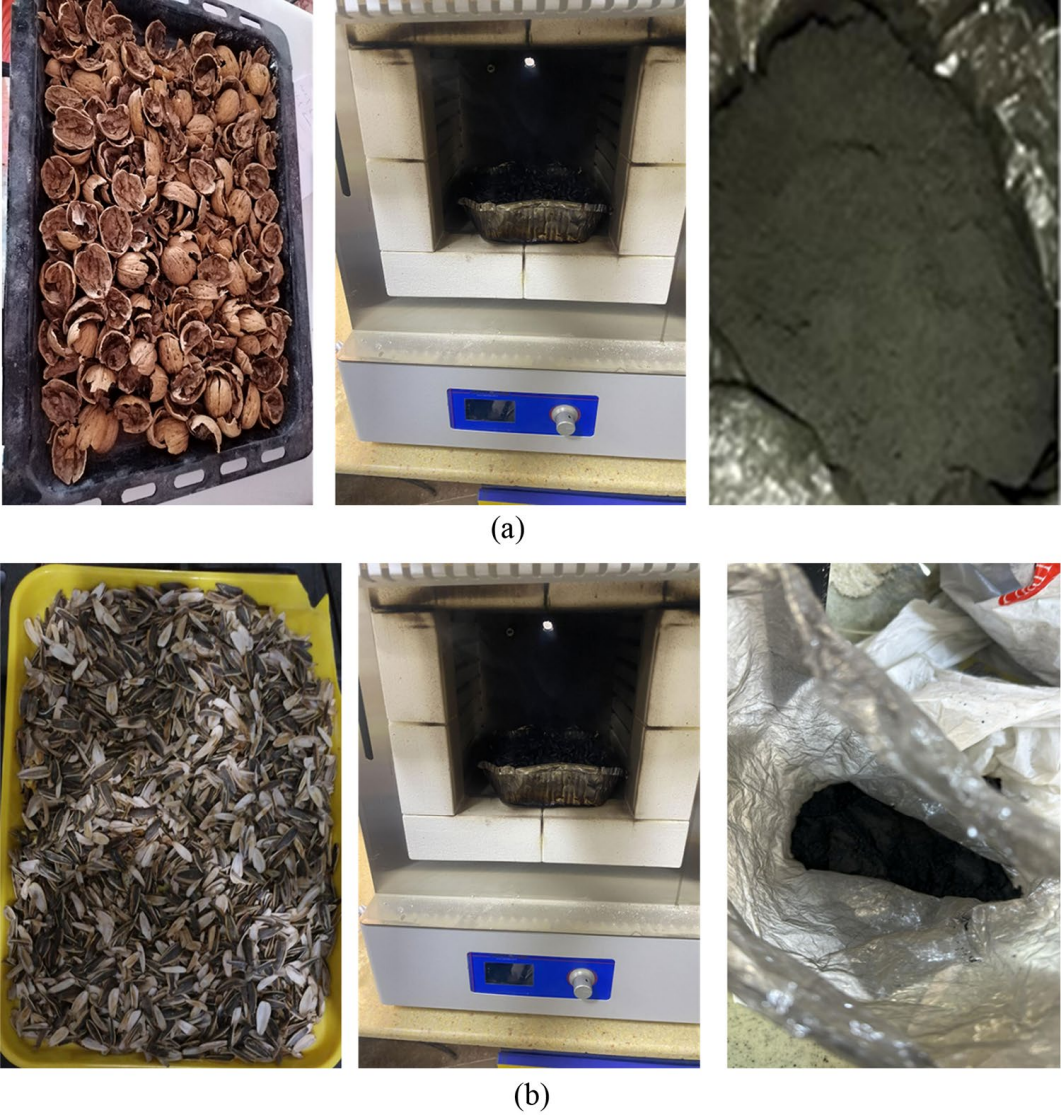
Ashes used: (**a**) Nano walnut shells ash; (**b**) Nano sunflower ash.

**Figure 4:**
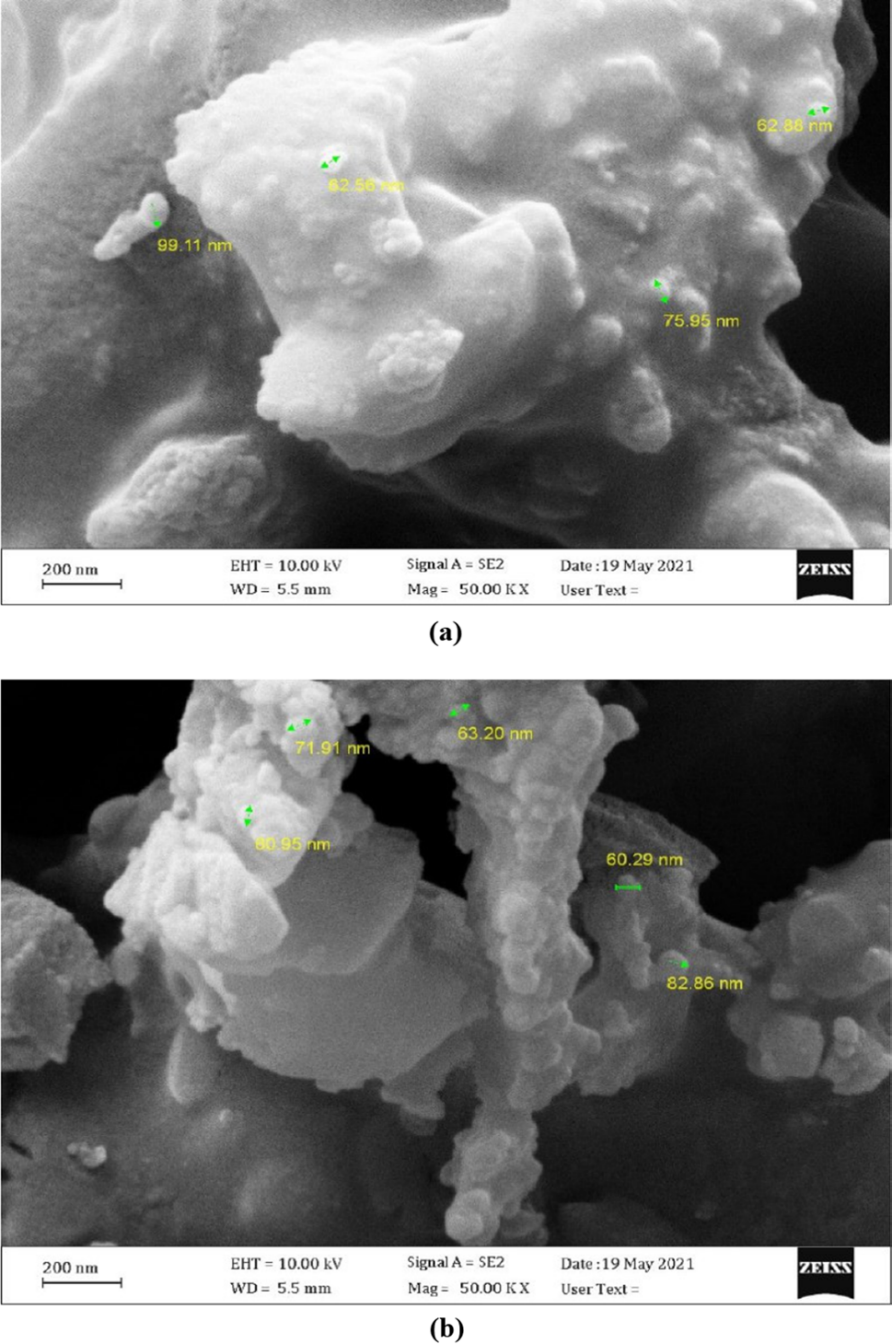
SEM micrographs of fine particles: (**a**) Nano sunflower ash; (**b**) Nano walnut shells ash.

**Figure 5:**
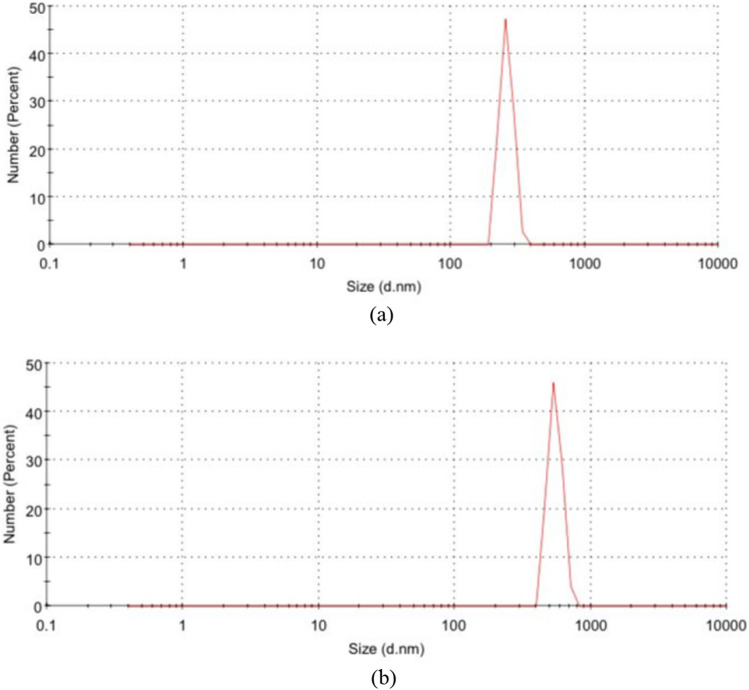
Particle size distribution: (**a**) NSFA (**b**) NWSA.

**Figure 6:**
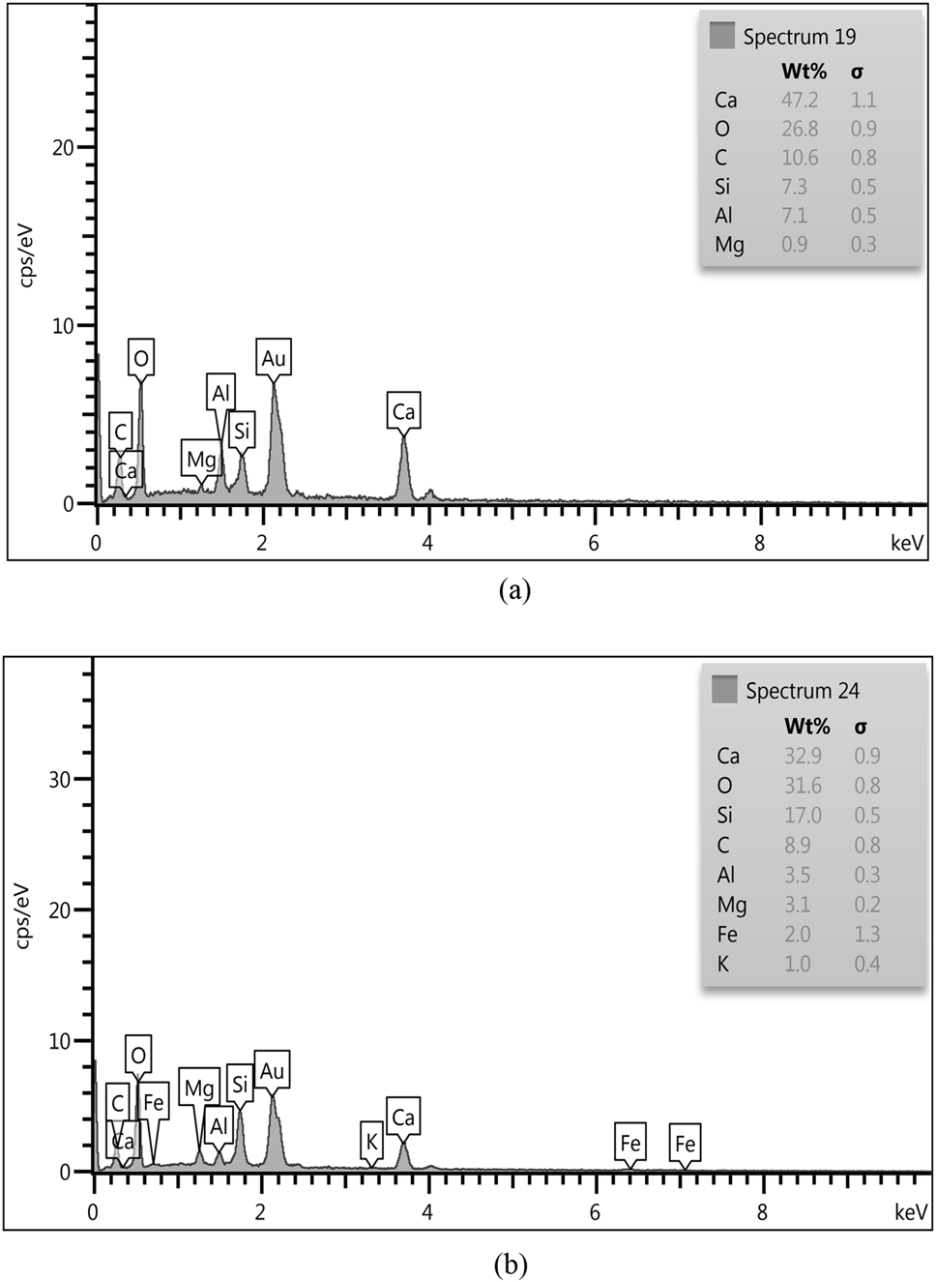
EDS Analysis. (**a**) Nano sunflower ash. (**b**) Nano walnut shells ash.

In the current research, the walnut shells ash (WSA) and sunflower ash (SFA) were acquired from the northern regions in Iraq. The WSA and SFA were put in oven at a 700 ± 50 °C for two hours according to the literature^51^. The ashes were cooled in an open area at room temperature for 30 min after being heated. After that, the ashes were crushed and sieved, then the particles passed through a 300 µm sieve in order to obtain nano WSA and nano SFA where the sifted WSA and SFA was ground through the usage of a ball mill for 3 h. However, the rotation speed of the mill was 250 rpm with steel ball utilization and the ball to charge ratio in many situations it was 10:1, for effective ball milling conditions you can go up to 20:1. For a ductile charge it should be better with 15:1. In this study, the ratio of 15:1 was used. Figure 3 shows the visual inspection of NWSA and NSFA. The physical characteristics were tested by SEM analysis, which revealed that NSFA and NWNA are irregular and micro porous as illustrated in Table 2 and Fig. 4, respectively. The NSFA chemical characteristics and NWSA were determined by X-ray fluorescence (XRF) spectroscopy analysis as illustrated within Table 1. Scanning electron microscopy (SEM) confirmed that the average NWSA and NSFA particle size is 74 nm and 74.5 nm, respectively (Fig. 4). Furthermore, Fig. 5 illustrate the particle size distribution of NSFA and NWSA. EDS analysis of NSFA and NWSA is illustrated in Fig. 6.

ASTM C618^54^ presents chemical requirements and specifications for natural pozzolans for cement replacement. According to this specification, a material is considered as pozzolanic material when the sum of chemical components SiO_2_ + Fe_2_O_3_ + Al_2_O_3_ are higher than 70% and the percentage of sulfur is less than 4%. In this research, the summation of the chemical components for SiO_2_ + Fe_2_O_3_ + Al_2_O_3_ were 81.85%, 52.99% and 79.3% for waste ceramic powder (WCP), nano walnut shells ash (NWSA) and nano sunflower ash (SFA) respectively. According to the results, WCP and SFA are considered as pozzolanic materials due to the fact that the values are higher than 70.0%. In addition, the percentages of sulfur trioxide (SO_3_) in WCP, NWSA and SFA were lower than 4%.

#### Admixture

The Aqueous solution of modified Polycarboxylate-based superplasticizer studied in the current research was characterized by a significantly low water to cement (W/C) ratio according to ASTM-C494^55^ standard Types G and F. The SP content at 1.6% of cement weight was utilized in all mixtures after some trial mixes^56^.

#### Natural aggregates

**Figure 7:**
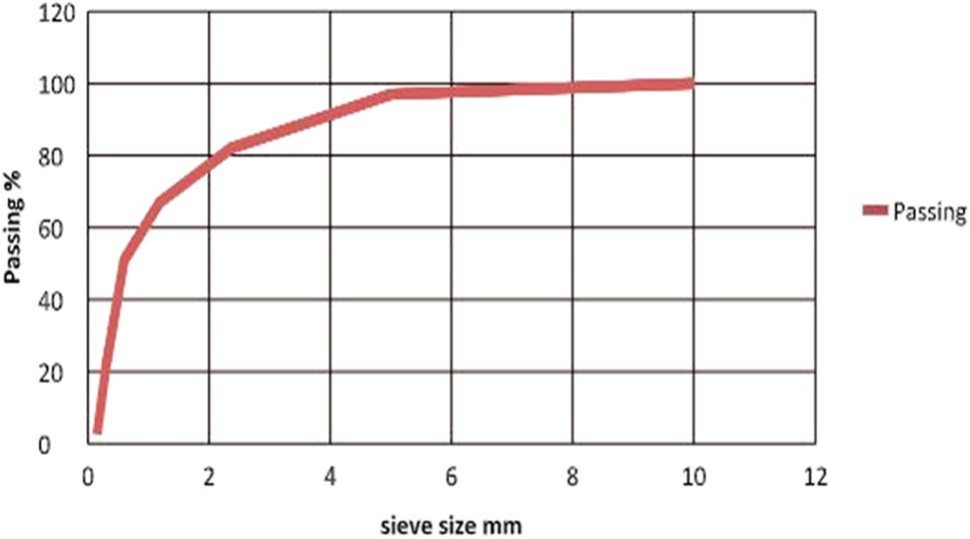
The Gradient of normal sand.

**Figure 8:**
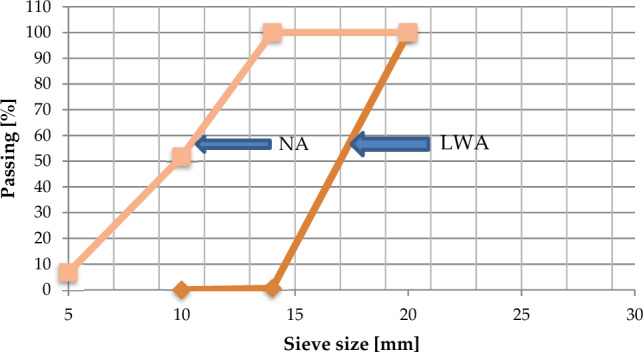
The Gradient of normal (NA) and lightweight aggregate (LWA).

In the current study, the local natural sand and river gravel were utilized as fine aggregate and coarse aggregates. Aggregates were examined following ASTM C33^57^ specification. Natural fine aggregate was utilized in this research from the Al-Bokhirbeet region. Figure 7 illustrates the sand grading that was found in zone two. However, Fig. 8 illustrated the river gravel gradient (normal aggregate). The sand physical characteristics were as follows; specific gravity, fineness modules, absorption, and sulfate content were 2.66, 2.8, 1%, and 0.21%, respectively. However, the river gravel physical characteristics were as follows; specific gravity, absorption, and sulfate content, are 0.04%, 0.3% and 2.7% respectively.

#### Plastic aggregates (lightweight aggregate)

**Figure 9:**
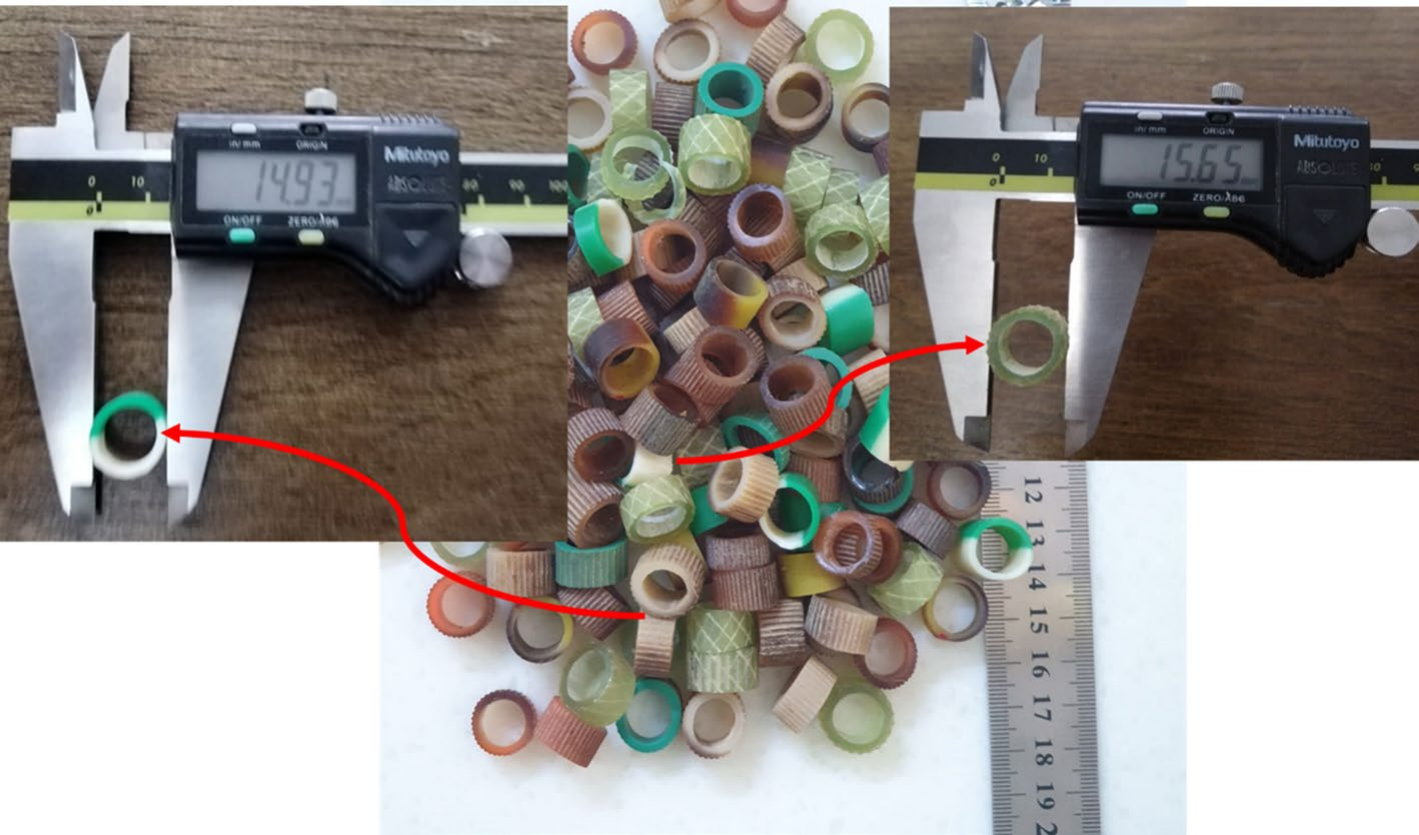
Lightweight waste plastic water hose manually cut using special scissors.

The waste plastic (Waste Polyvinyl chloride) was utilized in the current research by replacing constant recycled waste plastic percentage as Lightweight coarse aggregate by 75% of normal coarse aggregates within the mix for preparing concrete samples incorporated with plastic aggregates. The lightweight coarse aggregate was made from waste plastic water hose that were gathered and cut manually by special scissors. The procedure of preparing lightweight plastic waste aggregate is shown in Fig. 9. The two sizes of lightweight waste plastic aggregate are 15.65 mm and 14.93 mm. The lightweight aggregate particle size distribution of plastic waste is illustrated in Fig. 8.

### Methodology mixture proportions, mixing sequence

The lightweight self-compacting concrete (LWSCC) mixes proportions are shown in Table 3. The LWSCC mixes design were 0.34 of water-to-binder ratio (w/b). The cementitious materials total mass was designed at the value of 500 kg/m^3^ and its 25% percent was added by waste ceramic powder. The following parameters were kept constant: SP content as 3% of cement content, the waste plastic replacement percentage as 75% of normal coarse aggregates and the ratio of fine to coarse aggregates as 1:1.3. Seven LWSCC mixes with various partial substitution for cement by ashes from the nano walnut shells ash and nano sunflower ash combustion were prepared (0, 10, 20, and 30%).

**Table 3:** Concrete mixes proportions (kg/m^3^).

Mix ID	Binder content		NSFA^1^	NWSA^2^	W/b^3^	Sand	Coarse aggregate		Water	SP^6^
	Cement	Ceramic powder					NWA^4^	LWA^5^		
Control	400	100	–	–	0.34	820	234	310	170	8
10 NSFA	350	100	35	–	0.34	820	225	310	170	8
20 NSFA	310	100	63	–	0.34	820	225	310	170	8
30 NSFA	280	100	84	–	0.34	820	225	310	170	8
10 NWSA	350	100	–	35	0.34	820	225	310	170	8
20 NWSA	310	100	–	63	0.34	820	225	310	170	8
30 NWSA	280	100	–	85	0.34	820	225	310	170	8

^1^NSFA: Nano sunflower ash.

^2^NWSA: Nano walnut shells ash.

^3^W/b: Water/binder (cement + waste ceramic powder).

^4^NWA: normal weight aggregate (river gravel).

^5^LWA: lightweight aggregate (waste plastic).

^6^SP: Superplasticizer.

The mixing steps were started by mixing the waste plastic as lightweight coarse aggregate, normal coarse, and fine aggregates for about two minutes then the powders which consisted of cement and ceramic powder were added to aggregates in the mixer and mixed/rotated an extra one minute. After that, one-third of the water was added to the mix and rotated for about two minutes. Consequently, the remaining water was mixed with superplasticizer then added to the mixer and rotated for about three minutes before resting for one minute. Finally, the mixer was rotated for a further two minutes.

#### Fresh properties

**Figure 10:**
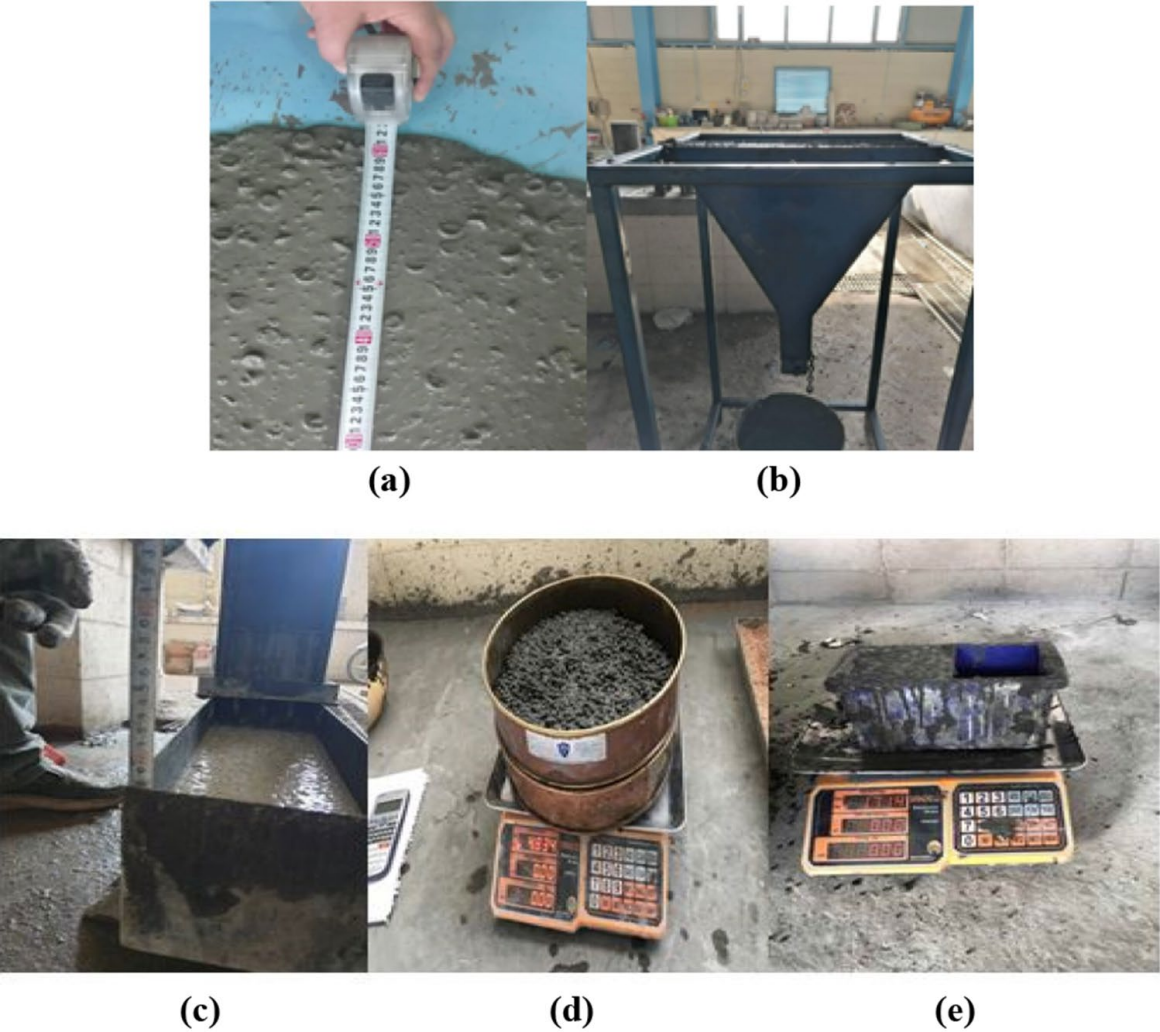
Fresh properties of LWSCC: (**a**) Slump flow; (**b**) V-funnel; (**c**) L-box; (**d**) Segregation; (**e**) Unit weight.

In order to describe fresh concrete characteristics, several terms have been utilized, such as finish ability, compatibility, pump ability, mobility, flowability, and consistency where workability is often utilized for defining some of those modern concrete characteristics. The calculation for lightweight self-compacting concrete (LWSCC) filling capacity with and without waste nano sunflower ash and nano walnut shells ash were carried out by fresh density (kg/m^3^), segregation analysis, L-box testing, V-funnel (sec), T500 (sec), and slump flow (mm) testing. For fresh concrete passing ability and the viscosity for remaining homogeneous in composition, it was determined in its fresh state and a calculation was carried out by segregation resistance testing. All the fresh concrete tests were conducted by utilizing EFNARC standards^58^ as illustrated in Fig. 10. In addition, fresh unit weight test was conducted for all LWSCC mixes.

#### Hardened properties

##### Mechanical properties

**Figure 11:**
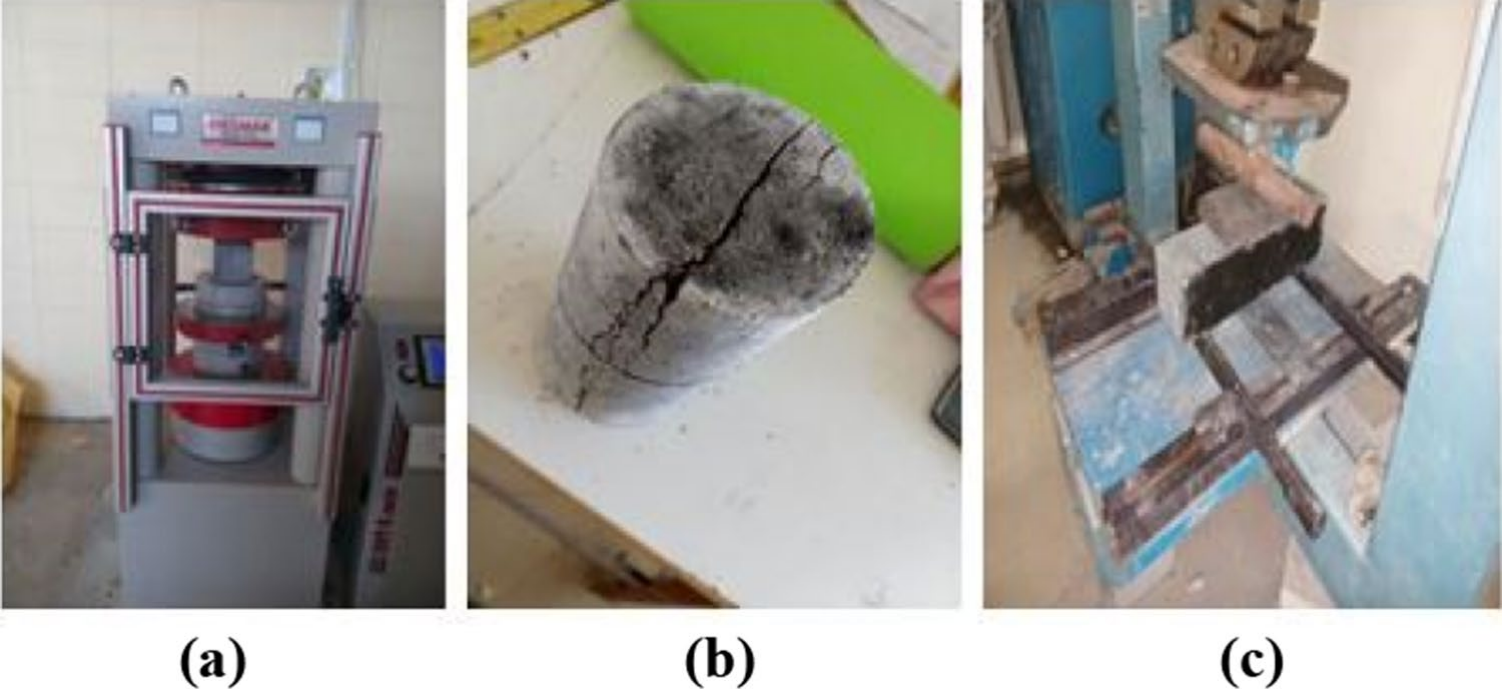
Testing hardened properties: (**a**) compression test; (**b**) splitting tensile strength; (**c**) flexural strength.

Three preparations of samples were carried out for determining each characteristic. Samples of all lightweight self-compacting concrete (LWSCC) mixes were compacted and cast through the use of vibrating table. All samples were removed from the molds 24 h after the cast procedure then placed in a water tank for 28 days at a temperature of 20 ± 5 °C. Figure 11 illustrates the hardened characteristics test of the samples. The hardened concrete samples characteristics were tested as the following:

- The test of compressive strength was conducted on cube samples with an edge length of 15 cm with a constant loading rate of 0.6 MPa/s depending on ASTM C39^59^ standard at ages corresponding to 7, 14, 28, and 60 days.
- The test of splitting tensile strength was carried out on cylinder samples with dimensions of 100 × 200 mm by loading the same with a constant rate of 0.9 MPa/min according to ASTM C496-C^60^ at the ages of 7, 14 and 28 days.
- The test of flexural strength was conducted on prism samples with dimensions of 100 × 100 × 500 mm by loading the same with a constant rate of 1 MPa/min according to ASTM C78-15a^61^ at the ages of 7, 14 and 28 days.

##### Durability properties

**Figure 12:**
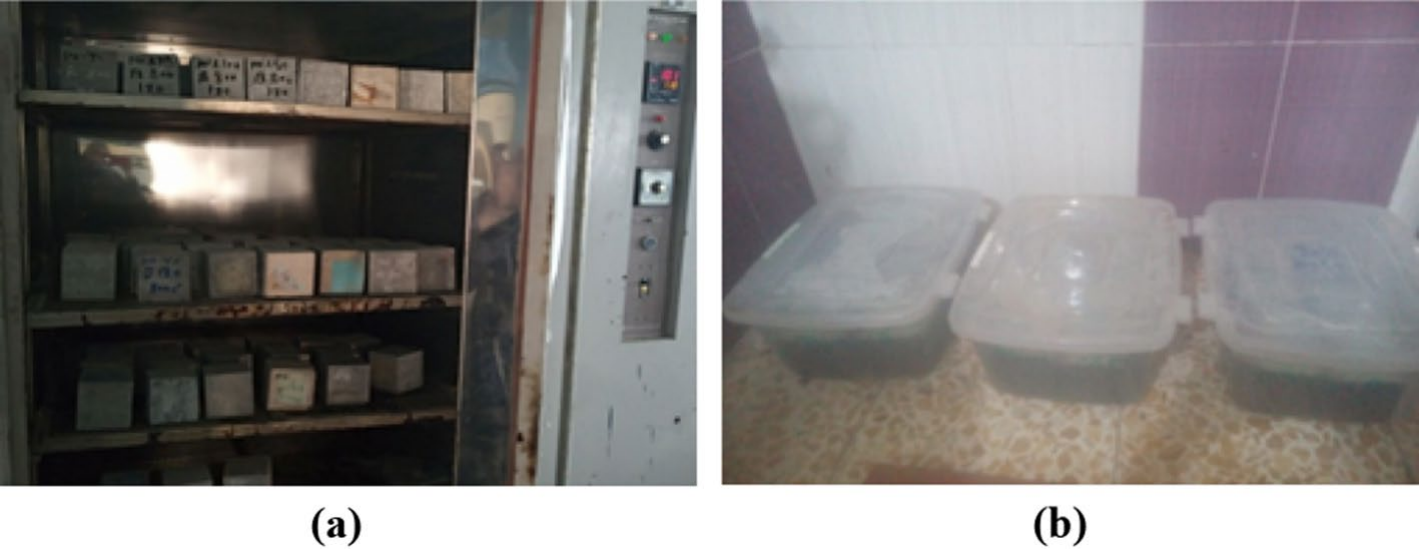
(**a**) Cubes exposure to evaluated temperature; (**b**) cubes immersed in water containing 10% MgSO_4_ of water weight.

The characteristics of durability were taken into consideration by measuring compressive strength and dry density for specimens' dimension 100 × 100 × 100 mm after exposing to elevated temperature at 800 °C for 3 h with a rate of 50 °C/min. Also, other samples were immersed within magnesium sulfate concentration (MgSO_4_) at 10% of water weight. The tests for the concrete sample’s durability characteristics were as the following:

- The tests of dry density and compressive tests were carried out on the specimens after exposing to temperature up 800 °C for 3 h with 50 °C/min rate. The specimens were dried in the furnace at 110 °C for 24 h. The mass was initially recorded, before exposure to a specified degree of burning after which the samples were cooled at room temperature. After that, the mass calculation was conducted in order to measure the loss in mass. Also, the compressive strength and the dry density for the specimens were measured. Figure 12 shows the burning test process.
- The test of resistance to sulphate attack was conducted for studying the impact of nano sunflower ash and nano walnut shells ash as a partial substitution with various percentages (10, 20 and 30% by weight of cement). The 100 × 100 × 100 mm concrete cube samples were cured and cast within water for 28 days. The preparation of magnesium sulphate (MgSO_4_) solutions with initial concentration of 10% by water weight was conducted within acid resistant tanks. Then specimens were extracted from the curing tank after finishing the curing procedure and were cleaned in order to remove the loose materials before the test process. After that, the samples were immersed into sulphate solution for 30 days as illustrated in Fig. 12. The solution was renewed weekly in order to preserve a constant concentration. After immersing for 30 days, the LWSCC sample unit weight and compressive strengths was found and the calculation of percentage change within the strength was carried out. Consequently, the mixture which showed less strength loss after sulphate attack^62^ was determined and it was taken into consideration as an optimum mixture.

## Results and discussion

### Fresh properties of LWSCC

Table 4 illustrates the results for the lightweight self-compacting concrete (LWSCC) fresh characteristics. V-funnel tests, T50 and Slump flow were utilized in measuring the LWSCC filling capability. However, L-box was utilized in measuring the LWSCC passing capability. Also, GTM screen stability was utilized in measuring the LWSCC segregation resistance. Table 4 illustrates the LWSCC results for wet unit weight.

**Table 4:** LWSCC's fresh concrete characteristics.

Mix ID	Slump flow (mm)	T50 cm (s)	V-funnel flow time (s)	L-box (h_2_/h_1_)	GTM Screen stability test (%)	Unit weight (kg/m^3^)
Control	780	2.5	6	1	16	1850
10 NSFA	730	4	9	0.9	12	1820
20 NSFA	680	4.5	12	0.8	10	1800
30 NSFA	630	5	15	0.7	8	1780
10 NWSA	730	5	10	0.9	13	1810
20 NWSA	710	6	8	0.85	11	1780
30 NWSA	690	7	12	0.8	9	1750
Range (EFNARC)	650–800	2–5	6–12	0.8–1	0–15	–

#### Slump flow diameter, slump flow time (T50) and V-funnel flow time

**Figure 13:**
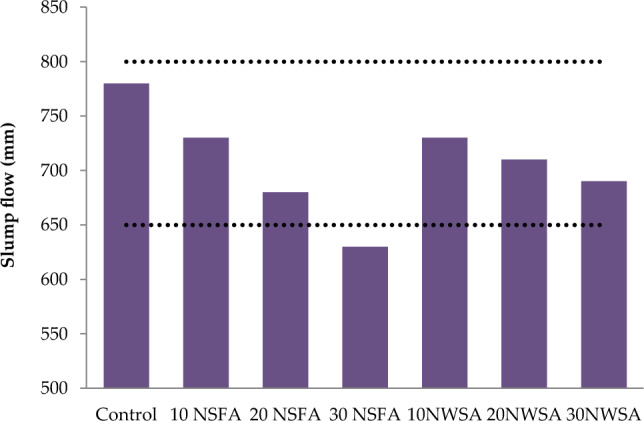
Slump flow test results of mixtures.

**Figure 14:**
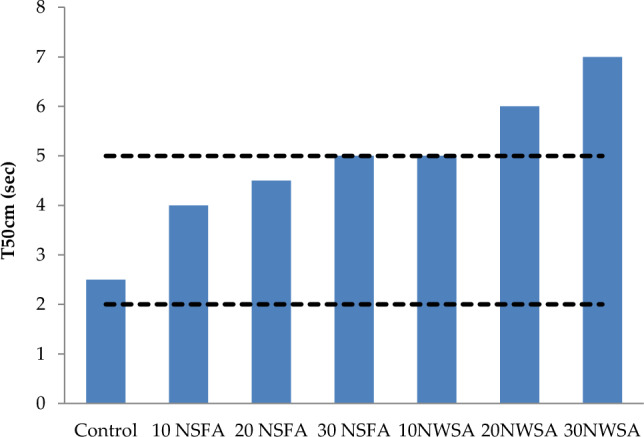
Slump flow time (T50 cm) test results of mixtures.

**Figure 15:**
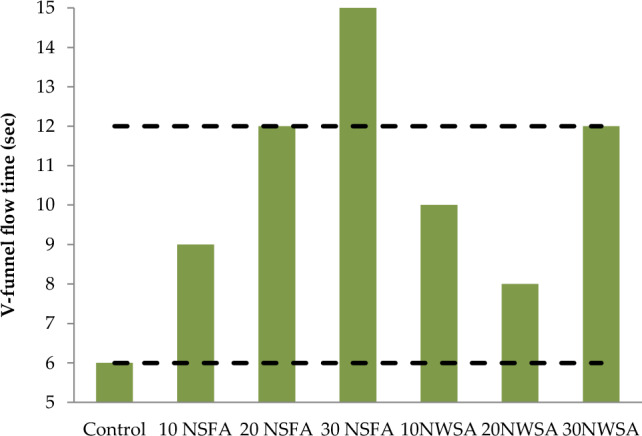
V-funnel flow time test results of mixtures.

Figures 13, 14, 15 illustrate the results of V-funnel, T50 and slump flow for the LWSCC mixture, respectively. Generally, the results coincided with the EFNARC results^58^ for all mixes excluding the one with 30 nano sunflower ash (NSFA) at V-funnel flow time and slump flow test. However, concrete specimens including 30% of NSFA and 20% of NSFA exceeded the EFNARC limit at slump flow time (T50) where the slump flow was from 780 to 630 mm. The T50 results of the mixtures were allocated between 2.5 and 7 s while the results of V-funnel were between 6 and 15 s. A comparison with the control mixture illustrated that filling capability was reduced when NWSA and nano walnut shells ash (NWSA) were utilized as the substitution ratios for content of cement. The low workability was possibly because of the high specific surface areas appearance that was around six times of those within the cement mixture and the mixtures characterized by irregular shapes as illustrated within Fig. 5. The results coincided with previous studies which investigated the impact of the various pozzolanic materials percentages usage on LWSCC filling capability^22–27,63^. Furthermore, when increasing the contents of NWSA and NSFA, the binder volume fraction and specific surface area are also raised due to the adsorbed water large quantity and the high surface area and the free water amount within the mortar was reduced^63^. The decrease in the flowability of self-compacting concrete (SCC) was also reported by Subası et al.^64^ when 20% waste ceramic was used as a substitute for cement. Furthermore, the current findings align with the research conducted by Agwa et al.^63^, which reported a decrease in the filling capability of LWSCC when rice straw ash and cotton stalk ash were utilized as substitution ratios for cement content. This decrease can be attributed to the presence of high specific surface areas, which are approximately six times greater than those found in the cement mixture and mixtures with irregular shapes.

#### L-box height ratio

**Figure 16:**
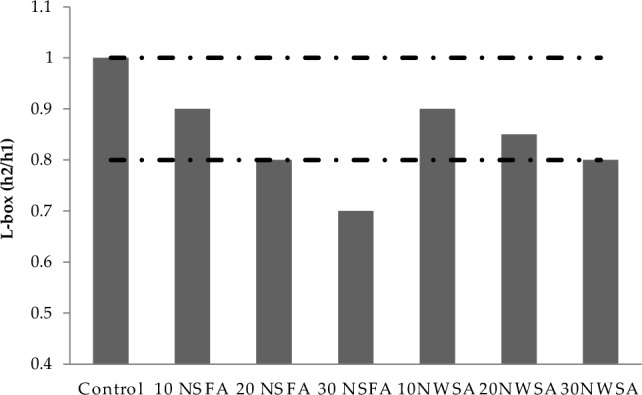
L-box test results of mixtures.

Figure 16 illustrates the results of LWSCC mixtures passing capability for L-box. The use of NWSA and NSFA within LWSCC decreases the passing capability in contrary to that within the control mix. The results of L-box acquired from (h_2_/h_1_) ratios began from 0.9 to 0.80 for 10% to 30% of NSFA, respectively. Similarly, the results of L-box began from 0.9 to 0.8 for 10% to 30% of NWSA, respectively. The L-box results of LWSCC mixtures coincided with EFNARC^58^ excluding that for 30NSFA. Dolatabad et al.^42^ reported similar findings in their investigation, whereby nanoparticles were utilised as a cement substitute in LWSCC. The authors' conclusion was that the inclusion of nanoparticles in the control mix resulted in a reduction in the passing ability. This finding may be attributed to an increase in the concentration of nanoparticles, resulting in a decrease in the presence of free water and an enhancement in particle packing, thus leading to a rise in internal friction.

#### Segregation resistance

**Figure 17:**
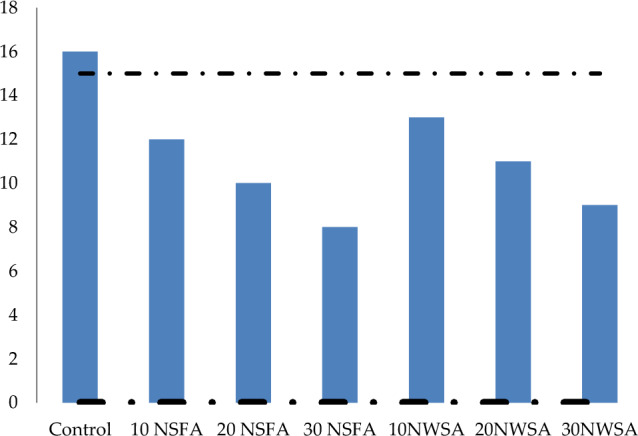
Mixture stability tests performed with GTM Screens.

Segregation resistance (GTM screen stability) was utilized in measuring the LWSCC segregation resistance. Figure 17 illustrates the results for test values of GTM screen stability which was between 16 and 9%. With the exception of the control mix, all of the GTM screen mixes tested were within the acceptable range in terms of screen stability.

In the same way, Hani et al.^45^ observed a decrease in segregation resistance as the quantity of nanosilica increased. Nevertheless, it is important to note that the results fell within the anticipated range. The decrease can likely be attributed to the nanosize and finer particles of NSFA and NWSA in comparison to cement. Thus, the inclusion of NSFA and NWSA leads to an increase in both the surface area and volume fraction of the binder. This increase in surface area results in a greater adsorption of water, thereby potentially increasing the requirement for superplasticizer^65^.

#### Fresh density

**Figure 18:**
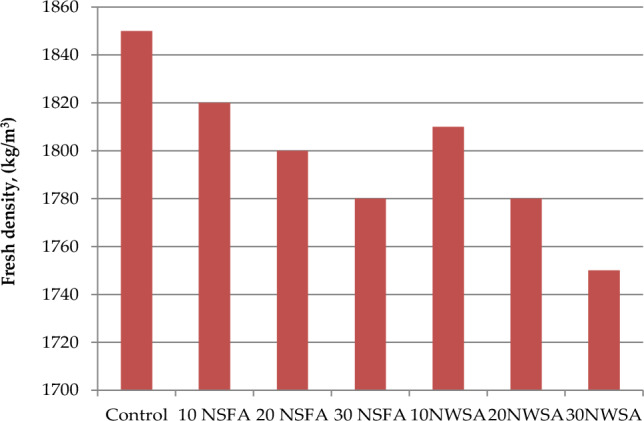
Fresh density test results of mixtures.

Figure 18 illustrates the nano sunflower ash (NSFA) and nano walnut shells ash (NWSA) mixed with LWSCCs fresh density. Mainly, the LWSCC fresh density was impacted because of several parameters such as content of cementitious additives, aggregates, and cement specific gravity. Nevertheless, the specific gravity was the main source for affecting the LWSCC workability and flowability. In this research, the cement content, superplasticizer content, fine aggregate content, coarse, and water-to-binder ratio (w/b) were kept constant. The results illustrated a decrement within the fresh density with NWSA and NSFA. The NWSA and NSFA addition gradually decreased the LWSCC samples fresh density. Furthermore, the highest fresh density value of 1850 kg/m^3^ was acquired by the control sample. However, for LWSCC 30% NWSA reported the lowest value of 1750 kg/m^3^. The reduction within the density was because of the NSFA particles specific gravities results of (3.2 g/cm^3^) and NWSA (2.17 g/cm^3^). Thus, raising the amount of NWSA and NSFA lead to a reduction in the LWSCC mixes fresh density. Besides, Du et al.^66^ reported that the nano materials addition reduced fresh concrete density (fresh density) due to C-S–H under specific quantity of nano materials. Another reason for that was illustrated in Table 4 where the cement amount was partially substituted by NWSA and NSFA where that decrement lead to a reduction in the mixture’s fresh density^33,34,67^. Therefore, the previous arguments are considered as an explanation for lower fresh density for all the mixtures.

### Hardened properties of LWSCC

Table 5 illustrates the results for the LWSCC hardened characteristics. Flexural strength, splitting tensile and compressive tests are utilized in measuring the LWSCC mechanical characteristics and LWSCC test dry unit weight are utilized in measuring physical characteristics. However, the durability characteristics were measured by calculating LWSCC specimens’ unit weight and compressive strength after exposing specimens of LWSCC to elevated temperature and sulphate attack.

**Table 5:** Hardened concrete properties of LWSCC.

Mix ID	Dry density (kg/m^3^)	Compressive strength (MPa)				Tensile strength (MPa)			Flexural strength (MPa)		
		7 days	14 Days	28 Days	60 days	7 days	14 days	28 days	7 days	14 days	28 days
Control	1820	22	27	31	35	2.0	2.7	2.9	2.6	3.0	5.2
10 NSFA	1780	20	25	29	32	1.2	1.9	2.8	1.88	2.95	4.3
20 NSFA	1760	15	19	25	29	1.6	2.0	2.4	2.25	3.28	4.6
30 NSFA	1740	14	18	21	24	1.1	1.75	2.0	2.33	2.75	3.9
10 NWSA	1780	22	26	30	33	1.95	2.8	3.0	3.66	4.1	5.2
20 NWSA	1750	18	22	28	30	2.0	2.3	2.7	3.0	3.5	4.6
30 NWSA	1720	16	20	26	29	2.1	2.35	2.5	3.26	4.0	4.5

#### Dry density

**Figure 19:**
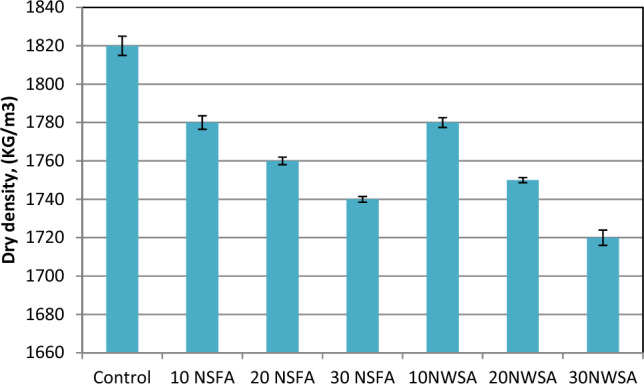
Dry density test results of mixtures.

The LWSCC hard density findings at 28 day curing age are illustrated in Fig. 19. The values are between 1820 and 1720 kg/m^3^ for 28 day curing age. The results showed that as the NWA and NSA content increased, the hard density reduced. This could be explained because of nano materials particles accumulation at higher contents^68^. Also, due to the nano materials higher surface area that caused an increment in the nanoparticles probability to agglomerate^69^.

### Mechanical properties

#### Compressive strength

**Figure 20:**
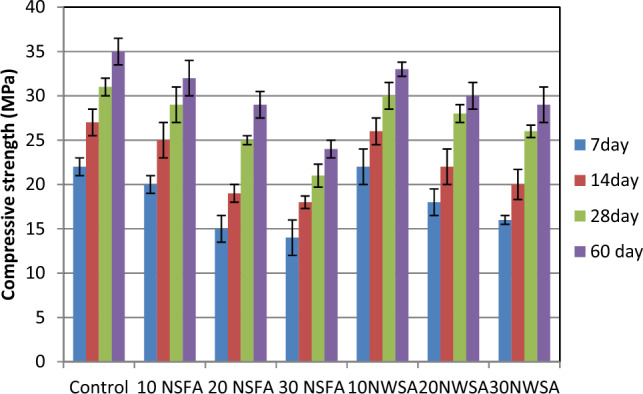
Compressive strength test results of mixtures at different ages.

Figure 20 shows the LWSCC mixes compressive strength on the 7, 14, 28, and 60 days. The reduction in compressive strength was observed as the NSFA and NWSA content increased. The NWSA and NSFA usage as a substitution for content of cement has a high negative impact on compressive strength, specifically at the 20% and 30% substitutions ratios for NSFA and at the 30% for NWSA. The slight reduction ratios within this study are 6.45%, 3.22% and 9.67% for the mixtures with 10 NSFA, 10 NWSA and 20 NWSA, respectively. The explanation for that was reported by Rong et al.^68^ who figured out that the compressive strength increased with up to 3% nano silica content but then reduced at higher nano silica quantity. This decrement was due to the nano silica particles accumulation at higher contents and because of the nano silica higher surface area which raised the nanoparticles probability to agglomerate^69^. Another possible explanation for the decrease in compressive strength with the addition of NSFA and NWSA quantities is the substantial amount of accessible silica that reacted with all the calcium hydroxide created in the wet cement. Due to the inability of such a large volume of silica to react chemically with other materials, it is not regarded an acceptable outcome according to this study^14,22,27,70^. Furthermore, there are two factors that contribute to the strength of pozzolanic materials: the proportion of calcium oxide and the percentage of silicate oxide (SiO_2_) in the pozzolanic mixture. NWSA possess a lower CaO (50%) quantity compared to NSFA (13%)^59^. Eventually, the mixtures with NWSA possess slightly higher splitting tensile strength compared to those with NSFA.

#### Splitting tensile strength

**Figure 21:**
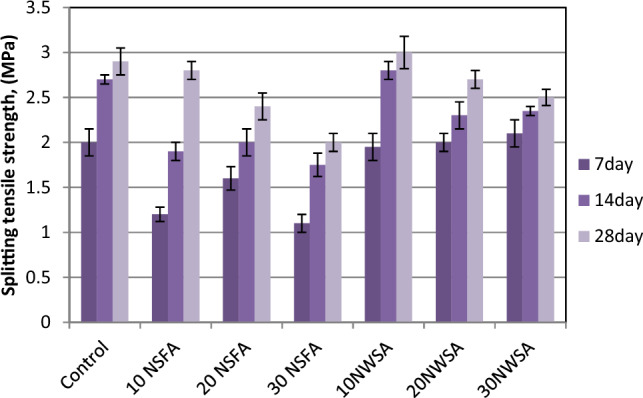
Splitting tensile strength test results of mixtures at different ages.

Figure 21 illustrates the findings of splitting tensile strength test for 7, 14 and 28 days specimens. The mix without pozzolanic materials (NWSA and NSFA) possessed the highest splitting tensile strength in comparison with the other mixes excluding 10NWSA where 10NWSA altered concrete has 3.44% slightly more than control mix. The explanation for that could be due to the pore structure enhancement in the cement paste caused by 10% NWSA substitution. Clearly, the increment of content of NSFA and NWSA caused a decrement within the splitting tensile strength in comparison with the control mix without NSFA and NWSA. Besides, the replacement of LWSCC samples with various NWSA percentages showed better results in comparison with samples included various NSFA percentages. This could be explained by the small NWSA surface area in comparison with NSFA surface area that caused the development of more hydrated products through the acceleration of cement hydration procedure^14,22,27,71^.

#### Flexural strength

**Figure 22:**
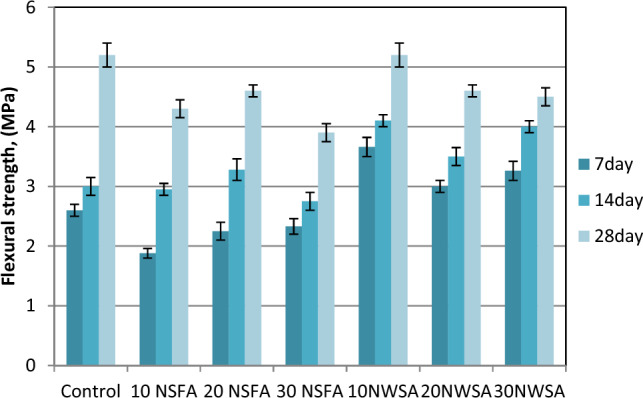
Flexural strength test results of mixtures at different ages.

Table 5 shows the results of flexural strength for all LWSC mixes without and with various NSFA and NWSA percentages. The 7, 14 and 28-days flexural strength of mixes is shown in Fig. 22. The flexural strength values range in the current research at 28 days were around 3.9–4.3 MPa and 4.5–5.2 MPa for samples including various NFSA and NWSA percentage, respectively however the flexural strength for control sample was 5.2 MPa. Obviously, flexural strength analysis illustrated a decrement with 10%, 20% and 30% NSFA substitution and with 20% and 30% NWSA. However, the NWSA mix strengths with 10% NWSA substitution are similar to the plain sample. The factor which might negatively impact the concrete flexural strength including NSWA and NSFA is the decrement within the flexural strength while raising the nanoparticles quantity which could be due to the nanoparticles distance at high quantities. By increasing nanoparticles quantity, the distance between them reduced which left a lower space for the Ca(OH)_2_ crystal growth which caused a lower C–S–H gel formation and thus a weaker concrete matrix microstructure, and therefore a lower flexural strength^72^. Besides, the same reasons were explained at section "Dry density".

#### Correlation between different Hardened properties

**Figure 23:**
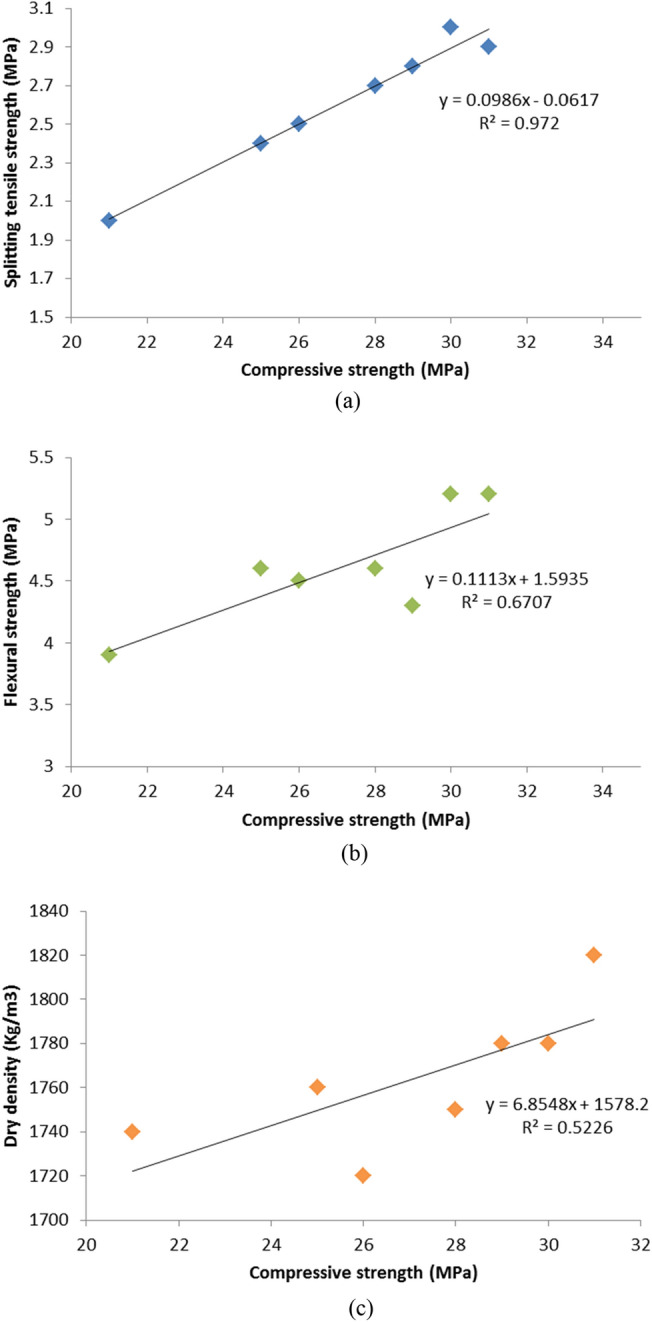
Relationship between compressive strength; (**a**) splitting tensile strength, (**b**) flexural strength, and (**c**) dry density.

This section focuses on the investigation of the correlation between various hardened characteristics. Figure 23 demonstrates a direct relationship between the compressive strength and (the dry density, flexural strength, and splitting tensile strength) of lightweight self-compacting concrete (LWSCC) when mixed with (MHA) and (WSA) as a substitution for cement within the LWSCC mix after 28 days. A reduction within compressive strength was typically accompanied by a decline in dry density, flexural strength, and splitting tensile strength. This relationship is supported by a strong correlation observed between compressive strength and splitting tensile strength values, with a high coefficient of determination (R^2^ = 0.972) as depicted in Fig. 23. However, the analysis revealed a suitable positive coefficient correlation of 0.67 was obtained for the relation between flexural strength and compressive strength, as shown in Fig. 22. On the other hand, a weak relationship was observed as 0.52 for the relation between dry density and compressive strength, as shown in Fig. 23.

### Durability properties

#### Elevated temperature

##### Dry density

**Figure 24:**
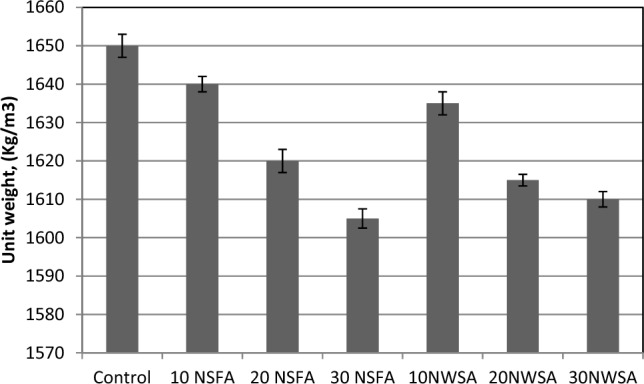
Unit weight test results of mixtures after exposure to 800 °C.

The various mixes densities within the hardened state after exposing to elevated temperatures at 800 °C with various NSFA and NWSA percentage within LWSC mixtures are illustrated in Fig. 24. Obviously, the concrete unit weight including NWSA and NSFA are inversely proportional to the substitution level and with elevated temperatures at 800 °C. The increment of NWSA and NFSA quantity in the mixes caused a decrement within the concrete unit weight. Besides, the usage of 10% NSFA and 10% NWSA substituted from cement weight within LWSCC caused a slight decrement within the concrete rate unit weight at 800 °C were 0.6% and 0.9%, respectively in comparison with reference SCC specimen. The maximum decrement within hardened density was acquired at high substitution levels with NSFA (30NFSA) and NWSA (30NWSA) at heating 800 °C with the value around 2.72% and 2.42% lower than that of reference concrete respectively.

The possible explanation for the decrease seen in the presence of increased NSFA (non-silica fine aggregate) and NWSA (non-waste silica aggregate) might be attributed to the NWSA and NSFA decomposition at elevated temperatures, resulting in the formation of pores within the matrix. These pores facilitate the removal of heat and exhibit effective behavior in the formation of cracking patterns. The heat produced in the LWSCC results in pore pressure, which subsequently leads to the spalling of the control specimen when exposed to a temperature of 800 °C. In the same way, it can be observed that 10NSFA exhibits a lower number of pores compared to both 20NSFA and 30NSFA. Consequently, due to its limited capacity to dissipate pore pressure, 10NSFA likewise experienced spalling at a temperature of 800 °C. The findings are consistent with the results reported by Ahsan et al.^73^, who observed a higher weight loss in modified seashell samples compared to control samples within the temperature range of 200–800 °C. On the other hand, the reduction in dry density in comparison with samples before exposure to temperature was 9.34%, from 7.86 to 7.75% and from 8.14 to 6.39% for control, from 10 to 30% NSFA and from 10 to 30% NWSA, respectively. This finding aligns with the results reported by Alobaidi et al.^74^, who noted that the use of nano-fly ash in self-compacting concrete (SCC) led to reduced weight loss in comparison to mixes containing fly ash and the control mixture. One possible explanation for this behavior is that the specimens containing different ratios of NWSA and NSFA exhibit reduced levels of free water compared to the reference collection. This may be attributed to the increased surface area of NWSA and NSFA at lower temperatures. The observed weight loss can be attributed to the loss of the free water that is trapped inside the capillary pores^75^.

##### Compressive strength

**Figure 25:**
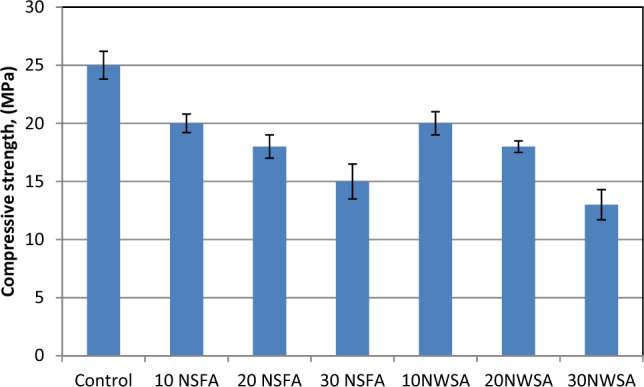
Compressive strength test results of mixtures after exposure to 800 °C.

Figure 25 demonstrated a systematic decrement within the compressive strength before and after exposing to elevated temperatures at 800 °C while increasing the content of NSFA and NWSA. At 28 days, the compressive strength was in the range of 20–15 MPa, and 20–13 MPa, for concretes including various percentage of NSFA and NWSA after exposing to elevated temperature, respectively. However, the reference concrete specimen was 25 MPa for samples after exposure to elevated temperature. Obviously, introducing NWSA and NSFA as a partial substitution for cement resulted in higher residual compressive strength loss. Nevertheless, the lowest increment within compressive strength values was observed with 10% NSFA and 10% NWSA. Besides, the decrement percentage for same sample 10% NSFA and 10% NWSA after exposing to elevated temperature were 20 and 20%. However, the highest decrement within compressive strength was 48% and 40% for 30% NSFA and 30% NWSA, respectively. Other researchers have previously reported similar results^75,76^. The decrease in strength is an inevitable consequence of the hydrothermal effect, which involves the loss of both absorbed and free water. Additionally, concrete undergoes numerous chemical and physical transformations, such as cement dehydration resulting in the shrinkage of the paste, voids, and formation of cracks. These alterations in the concrete matrix occur when it is subjected to high temperatures^77^. The increase in pore pressure up to 200 °C due to the removal of free and adsorbed water leads to a decrease in strength as a result of micro-cracking^78^. The primary decomposition of calcium hydroxide (Ca(OH)_2_) occurs within the temperature range of 400–600 °C. The decomposition of calcium silicate hydrate (CSH) gel occurs at temperatures exceeding 560 °C, leading to significant reductions in both stiffness and strength^79,80^. According to a previous study^81^, temperatures over 500 °C result in significant and irreversible transformations. Furthermore, the primary factor leading to degradation at a temperature of 600 °C is the thermal instability of the aggregates. This degradation is mostly attributed to the de-carbonation process of calcium carbonate (CaCO_3_), which takes place within the temperature range of 600–800 °C. In addition, the decrement within the compressive strength of LWSCC mixes at (200–800 °C) is due to interfacial bond break-down attributed to incompatible volume change between aggregate and cement paste while dehydrating and heating of the calcium-silica hydrate within cement paste^82^. As well, the reasons which were mentioned at section "Dry density". Lubloy et al.^83^ reported that a lower CaO/SiO_2_ ratio of the binder means higher relative residual compressive strength in concrete after a thermal load of 800 °C. In this study, this ratio was smaller for control samples particles compared to NWSA and NSFA particles as can be seen from Table 1.

#### Sulphate attack

##### Dry density

**Figure 26:**
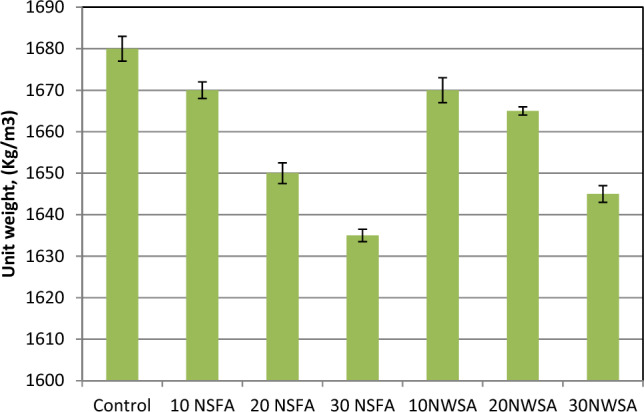
Unit weight test results of mixtures after exposed to 10% magnesium sulphate solution.

The findings of LWSCC hard density blended with NWSA and NFSA after sulphate attack are demonstrated in Fig. 26. The results are between 1670 to 1635 kg/m^3^, and 1670 kg/m^3^ to 1635 kg/m^3^ for (from 10 to 30%) NFSA and (from 10 to 30%) NWSA, respectively, with the LWSCC control samples result value of 1680 kg/m^3^. Raising the NWSA and NFSA amounts within the mixes caused a reduction within the concrete unit weight. The findings showed the increment of the NWSA and NFSA content lead to a reduction in the dry density. These findings are consistent with findings published by Siddique et al.^84^. The researchers noted that the magnesium sulphate utilized for simulating the sulphate attack on concrete generates magnesium hydroxide that lowers the pH of the solution, resulting in the production of ettringite and thaumasite within the pores of the concrete samples^84,85^. The samples mass grows when the voids become filled up owing to its formation. At the final stages of research, the magnesium sulphate solution produces a highly destructive impact on the hydration products within concrete. Due to the comparable ionic radii and valence of Ca + and Mg + ions, the reaction between magnesium sulphate and calcium silicate hydrate (CSH) results in the formation of silicate hydrate and brucite^86^. The process of breaking down polymeric calcium silicate hydrate (CSH) into its constituent components results in a reduction in mass.

However, samples including various proportions of NSFA and NWSA exhibited reduced percentages of density loss, ranging from 6.1% to 6.03% and 6.17% to 4.36%, respectively, as compared to specimens that were not subjected to Sulphate. Nevertheless, the control specimens had a drop of 7.6%. Concrete samples that incorporate NWSA and NFSA exhibit a notable increase in surface area, hence elevating the likelihood of nanoparticle agglomeration. Additionally, these specimens demonstrate diminished pozzolanic activity, leading to an increased presence of voids within the concrete. The presence of higher voids within the concrete matrix contributes to a delay in the breakdown of hydration products since these voids provide opportunity for the creation of ettringite^84^. Furthermore, it is notable that the decrease in density is less pronounced in specimens containing NWSA compared to specimens containing NSFA. This disparity may be related to the elevated alumina oxide content in the used pozzolana, exceeding 10% as indicated in Table 1. This higher alumina oxide concentration is responsible for the reduced sulphate resistance and increased mass loss^87^.

##### Compressive strength

**Figure 27:**
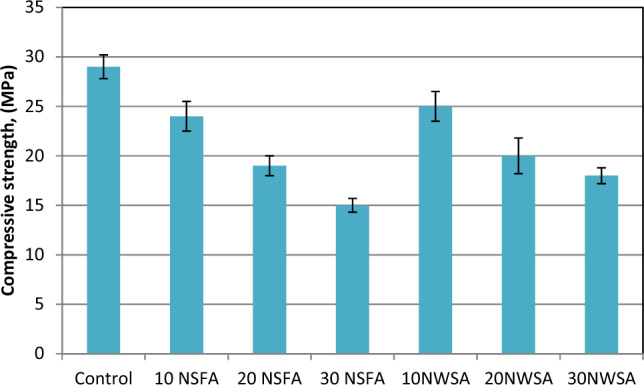
Compressive strength test results of mixtures after exposed to 10% magnesium sulphate solution.

The deterioration degree within concrete because of sulphate attack was measured by determining the residual compressive strength. Figure 27 demonstrates the residual compressive strength findings due to magnesium sulphate attack of LWSC samples with and without NWSA and NSFA. Obviously, increasing content of NWSA and NFSA reduces the NWSA and NFSA sulphate resistance mixed with LWSCC. The unblended LWSCC strength loss was less than those of NWSA and NFSA blended LWSCC. The reduction of compressive strength (%) for 10NFSA and 10NWSA LWSCC samples after sulphate attack in comparison with the samples results of specimens that was cured at tap water were 17.2% and 16.7% for 10NWSA LWSCC specimens, respectively. However, Strength loss (%) findings for LWSCC control sample were 6.5%.

As previously mentioned in Section "Dry density", the reaction between sulphate solution and CSH results in the formation of brucite and silicate hydrate. The brucite subsequently undergoes a reaction with silicate hydrate, resulting in the formation of magnesium silicate hydrate. This mixture exhibits minimal cohesive properties, hence resulting in a deficiency of inter-particle interaction within the concrete matrix^86^.

Furthermore, it may be hypothesized that the NWSA and NSFA samples, due to their much-elevated alumina oxide concentration, may exhibit a greater susceptibility to sulphate attack. In addition, it should be noted that the poor pozzolanic activity of NWSA and NSFA results in the reaction of Ca(OH)_2_ with sulphate, leading to the formation of gypsum and ettringite. This reaction ultimately causes expansions within the specimens. The process of expansion results in the cracking of samples; therefore, the strength is reduced^87^.

## X-ray diffraction analysis [XRD]

**Figure 28:**
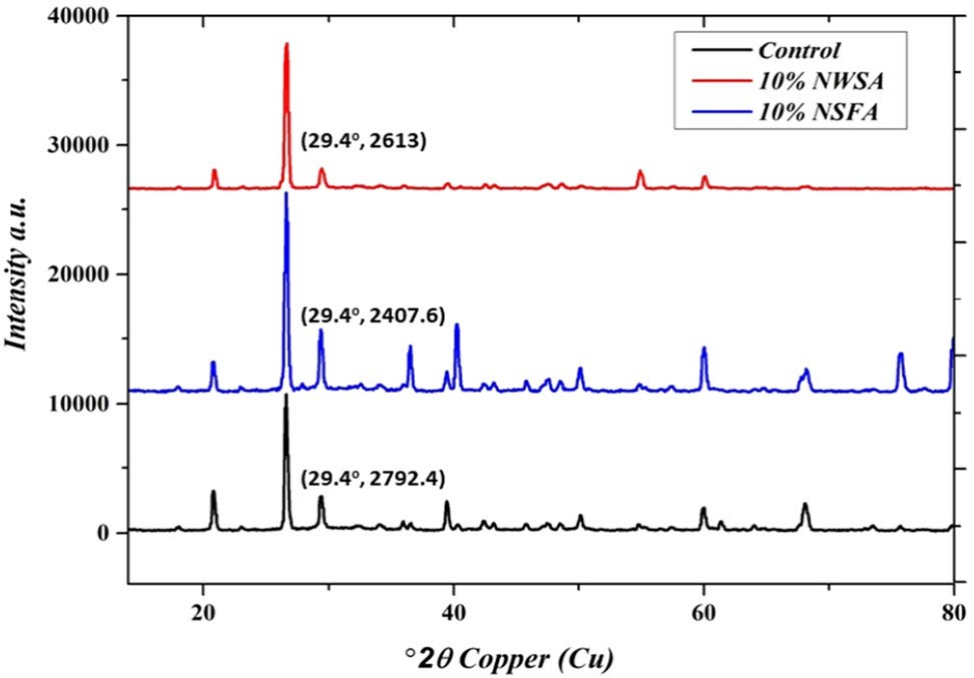
XRD patterns of control, 10% NSFA and 10% NWSA at 28 days.

The aim of carrying out XRD patterns is to illustrate changes in C–H intensity. It demonstrates production of C–S–H and consumption of CH crystal. Figure 28 illustrates the control of C–S–H for XRD patterns, 10% NSFA and 10% NWSA specimens at 28 days, respectively. The quartz (SiO_2_) high peaks are tracked at theta of 29.4°. As shown in Fig. 27, the C–S–H highest peaks are for the control specimens. Furthermore, adding 10% of both NWSA and NSFA reduced the intensity of C–S–H at 28 days. The XRD patterns results showed that the pozzolanic reactivity of 10% NWSA and 10% NSFA happened at 28 days.

## Conclusions

This study aimed to produce lightweight self-compacting concrete (LWSCC) by using nano sunflower ash (NSFA) and nano walnut shells ash (NWSA) as partial replacements for cement at levels of 0%, 10%, 20%, and 30%. Furthermore, a predetermined quantity of recycled waste ceramic powder will be used to replace cement, while a specified amount of waste plastic will be used to partially replace the normal coarse aggregate in all mixtures. In order to produce an environmentally friendly self-compacting concrete (SCC) structural material, with the aim of reducing the amount of waste deposited in public landfills. An analysis was conducted to examine the mechanical and durability characteristics of LWSCC (fresh and hardened properties). Based on the obtained data, the following conclusions were drawn:

- The results showed that mechanical milling walnut ash and sun flower ash increased their surface areas, but they did not operate as activators or fillers to aid hydration.
- In contrast to the control mixture, NWSA and NSFA substation ratios for cement content reduce workability. High surface area reduces workability. Thus, LWSCC workability requires more water.
- NWSA and NSFA decreased LWSCC fresh density because their particles have lower specific gravity than cement. NWSA and NSFA particles have a greater surface area, generating a matrix with more holes, reducing dry density. Agglomerating nanoparticles may explain this.
- The mixtures including 10% NFSA and 10% of NWSA slight reduced compressive strength compared to the control sample.
- Compressive strength decreased with NWAS and NSFA concentrations. Nano NWSA and NSFA higher concentrations lowered mechanical characteristics. Nanoparticle aggregation rises with NWSA and NSFA’s larger surface areas.
- The splitting tensile and flexural strengths decreased with increasing the ratios of NWSA and NSFA, while the flexural strength gave the same results as the control sample.
- All concrete mixtures lost significant compressive strength after 800 °C. LWSCC residual strength with NSFA was comparable to NWSA.
- With increasing NWSA content, dry density decreased marginally at higher temperatures. The LWSCC with NSFA had slightly reduced dry density loss than the control specimen. Little improvement in the NSFA pozzolanic reaction may explain reduced spalling, which is significant for thermal insulation.
- After soaking concrete in 10% magnesium sulphate, all mixes lost compressive strength. LWSCCs residual strength with varied NWSA percentages was slightly higher than similar ones. After sulphate attack, blends with 10% NSFA and 10% NSFA had higher residual compressive strengths than 21 MPa.
- The XRD data showed that NSFA and NWSA cement replacement lowered C–S–H at 28 days. NSFA reduced C–S–H more than NWSA.

The study's findings suggest that the mixes that include nano sunflower ash and nano walnut shell ash in varying proportions can be classified as structural lightweight concrete. This is supported by the compressive strength and dry density findings of the mixes, which fell between 1720 kg/m^3^ to 1780 kg/m^3^ and from 21 to 29 MPa, respectively. These values align with the specifications outlined in ACI 213 (2001) for structural lightweight concrete, which require a minimum strength of 17.0 MPa and a density ranging from 1350 to 1920 kg/m^3^. There are three distinct categories of lightweight concrete based on their strength levels: low-density concretes (0.7–2.0 MPa), moderate-strength concretes (7–14 MPa), and structural concretes (17–63 MPa). The density of these concretes falls between the ranges of 300–800 kg/m^3^, 800–1350 kg/m^3^, and 1350–≈1920 kg/m^3^, respectively.

## Recommendations

The authors suggest using EN 14,651 to find the residual flexural and tensile strengths. This test setup has the benefit of letting you precisely measure the crack width because the notch shows where the crack forms. The test is controlled and evaluated by measuring the crack width and load-crack mouth opening displacement (CMOD).

## Data Availability

The datasets used and/or analysed during the current study available from the corresponding author on reasonable request**.**
